# Suizide in Deutschland: Ergebnisse der amtlichen Todesursachenstatistik

**DOI:** 10.1007/s00103-021-03470-2

**Published:** 2021-12-28

**Authors:** Torsten Schelhase

**Affiliations:** H11 Gesundheitsstatistiken, Statistisches Bundesamt, Zweigstelle Bonn, Graurheindorfer Straße 198, 53117 Bonn, Deutschland

**Keywords:** ICD-10, Sterberate, Sterbealter, Suizidmethoden, Qualitätsverbesserung, ICD-10, Mortality rate, Age at death, Suicide methods, Quality improvement

## Abstract

Die Todesursachenstatistik ist die elementare Basis zur Ermittlung von wichtigen Indikatoren wie Sterbeziffern, verlorenen Lebensjahren und vermeidbaren Sterbefällen sowie deren Veränderungen im Laufe der Zeit. Ihre Ergebnisse fließen in die epidemiologische und medizinische Forschung ein und geben wichtige Hinweise für notwendige Präventionsprogramme und die Gesundheitspolitik im Allgemeinen.

In diesem Beitrag wird zunächst ein Überblick über die Historie, die Rechtsgrundlage und die Methodik der Todesursachenstatistik gegeben. Daran schließt die Darstellung der Daten zu Suiziden in Deutschland mit Schwerpunkt auf das Jahr 2019 an. Diese werden in Abhängigkeit von den Merkmalen Alter, Geschlecht, Region und Methode abgebildet. Vergleiche mit älteren vorliegenden Daten seit 1980 werden herangezogen. In einem Ausblick auf die Weiterentwicklung der Todesursachenstatistik werden die Gründe für aktuelle Schwachstellen im Ablauf der Datenerhebung und Lösungsmöglichkeiten aufgezeigt.

**Zusatzmaterial online:**

Zusätzliche Informationen sind in der Online-Version dieses Artikels (10.1007/s00103-021-03470-2) enthalten.

## Abstract (en)

The cause of death statistics is an elementary basis for determining important indicators such as death rates, years of life lost, and avoidable deaths as well as their changes over time. The results are used in epidemiological and medical research and provide important recommendations for prevention programs and for health policy in general.

This article first gives an overview of the history, legal basis, and methodology of the cause of death statistics in Germany. This is followed by the presentation of the data on suicides in Germany with a focus on the year 2019. These data are mapped based on the characteristics of age, gender, region, and method of suicide. Comparisons with older data available since 1980 are made. The outlook gives an overview of the further development of the cause of death statistics, the reasons for current weaknesses in the data collection process, and possible solutions.

## Einleitung

In Deutschland geschieht ca. jede Stunde ein Suizid, jeden Tag, an 365 Tagen im Jahr. Mit rund 1 % aller Todesfälle stellt der Suizid damit keine unbedeutende Todesursache dar, trotzdem wurde er in der Vergangenheit oft tabuisiert, zum Teil auch ignoriert. Informationen zu Suiziden und anderen Todesursachen entstammen vor allem der Todesursachenstatistik. Die Auswertung dieser Daten liefert wichtige Hinweise auf relevante Gesundheitsindikatoren wie Sterbeziffer, verlorene Lebensjahre und vermeidbare Sterbefälle. Sie ist Basis für die Todesursachenforschung, die die Zusammenhänge zwischen verschiedenen Einflussfaktoren wie Alter, Geschlecht, regionale Besonderheiten in der Sterblichkeit und diesbezügliche Veränderungen im Laufe der Zeit untersucht. Aus diesen Ergebnissen heraus werden Empfehlungen für kommende Handlungsfelder und Strategien für die epidemiologische Forschung, die Prävention und die Gesundheitspolitik abgeleitet. Ziel ist es, die Lebenserwartung und Lebensqualität der Bevölkerung durch präventive und medizinisch-kurative Maßnahmen zu erhöhen.

Dabei bietet die Todesursachenstatistik folgende Vorteile: Zum einen stellt die Kontinuität der Erhebung sicher, dass dem Nutzer Daten aus weit zurückreichenden Zeiträumen zur Verfügung stehen. Zum anderen werden die Daten entsprechend der Internationalen Statistischen Klassifikation der Krankheiten und verwandter Gesundheitsprobleme (ICD-10) der Weltgesundheitsorganisation (WHO) verarbeitet, was eine internationale Vergleichbarkeit ermöglicht. Letzter großer Pluspunkt ist die jährliche Vollerhebung aller Todesfälle.

Im Text wird ausschließlich der wertfreie Begriff „Suizid“ verwendet, grundsätzlich stehen für den Begriff des Suizids verschiedene andere Synonyme zur Verfügung: Die ICD-10 der WHO sieht als Bezeichnung „vorsätzliche Selbstbeschädigung“ vor, oft werden aber auch je nach Perspektive Begriffe wie Selbstmord, Selbsttötung oder Freitod gebraucht. In seinem Urteil vom 26.02.2020 [1] hat das Bundesverfassungsgericht im Zusammenhang mit dem sogenannten assistierten Suizid verdeutlicht, dass das allgemeine Persönlichkeitsrecht (Art. 2 Abs. 1 in Verbindung mit Art. 1 Abs. 1 Grundgesetz) ein Recht auf selbstbestimmtes Sterben umfasst. „Dieses Recht schließt die Freiheit ein, sich das Leben zu nehmen und hierbei auf die freiwillige Hilfe Dritter zurückzugreifen. Die in Wahrnehmung dieses Rechts getroffene Entscheidung des Einzelnen, seinem Leben entsprechend seinem Verständnis von Lebensqualität und Sinnhaftigkeit der eigenen Existenz ein Ende zu setzen, ist im Ausgangspunkt als Akt autonomer Selbstbestimmung von Staat und Gesellschaft zu respektieren.“ Damit wird auch unterstrichen, dass der Suizid strafrechtlich nicht mehr verfolgt werden kann.

In diesem Beitrag wird ein Überblick über die Ergebnisse der Todesursachenstatistik in Bezug auf Suizide in Deutschland gegeben. Dies beinhaltet nicht nur einen Überblick über die absoluten Zahlen, sondern auch eine nähere Betrachtung verschiedener Aspekte wie Suizid in Abhängigkeit vom Alter und Geschlecht. Neben der Darstellung der aktuell vorliegenden Ergebnisse des Berichtsjahres 2019 werden auch Zeitvergleiche abgebildet. Ergebnisse für das Berichtsjahr 2020 lagen beim Verfassen dieses Aufsatzes noch nicht vollständig und vollplausibilisiert vor. Auf die bis dato verfügbaren, vorläufigen Daten zum Berichtsjahr 2020, die im Zuge der Monatsberichte seit Juli 2021 veröffentlicht werden, wird daher nur kurz eingegangen.

## Die Todesursachenstatistik in Deutschland: Historie, Rechtsgrundlage und Methodik

### Historie

Die Angst vor ansteckenden Krankheiten war einer der wichtigsten Anlässe, Daten zu Todesursachen aufzuzeichnen [2]. Aus diesem Grund hat die Todesursachenstatistik eine recht lange Tradition, erste Aufzeichnungen für eine Auswahl von zum Tode führenden Krankheiten liegen seit der zweiten Hälfte des 19. Jahrhunderts vor, seit 1892 besteht eine „echte“ Statistik im Sinne einer umfangreichen Erhebung.

Mit der Umstellung des ersten ausführlichen Verzeichnisses für Deutschland auf die ICD der WHO Anfang der 1930er-Jahre wurden die Daten international vergleichbar. Daten, die in Anlehnung an die ICD generiert wurden, liegen in schriftlicher Form ab dem Jahr 1950 und in digitaler Form ab 1980 vor. Aufgrund der sich ständig ändernden Rahmenbedingungen (medizinischer Fortschritt, Veränderung der Arbeits- und Lebensverhältnisse etc.) wird die ICD regelmäßig angepasst. Seit 1998 wird in Deutschland die stark revidierte und erweiterte ICD in der Fassung der 10. Revision (ICD-10) angewendet. Die bereits entwickelte neue Version (ICD-11) wird in den nächsten Jahren in Deutschland umgesetzt.

Auch in der ehemaligen Deutschen Demokratischen Republik (DDR) wurde offiziell die ICD der WHO als Klassifikationsgrundlage verwendet. Politische Gründe haben jedoch dazu geführt, dass das Thema Suizid kleingehalten wurde [3]. Daher sind Vergleiche der Zahlen bis zum Jahr 1990 schwierig.

### Rechtsgrundlage

Der Gesetzgeber hat 1980 den geltenden Rahmen für die Todesursachenstatistik durch das „Gesetz über die Statistik der Bevölkerungsbewegung und die Fortschreibung des Bevölkerungsstandes“ (Bevölkerungsstatistikgesetz)^1^ geschaffen. Ziel des Gesetzes ist es, weitreichende Informationen zur bevölkerungspolitischen Lage zu erhalten, um darauf aufbauend mit Maßnahmen der Gesundheitspolitik und der Wissenschaft sowohl die allgemeine Sterblichkeit zu minimieren als auch die Lebenserwartung der Menschen zu erhöhen. In §1 des Gesetzes heißt es zum Zweck, dass für die Statistik der natürlichen Bevölkerungsbewegung unter anderem die Sterbefallstatistik einschließlich Todesursachenstatistik als Tatbestand erfasst werden soll (§1 1.d). Ein weiteres Ziel des Gesetzes ist, die Bestandteile der Bevölkerungsentwicklung und der Bevölkerungsverschiebung zu ermitteln (Begründung zum zuvor gültigen Gesetz vom 04.07.1957; Bundestagsdrucksache Nr. 3005 vom 12.12.1956).

### Methodik

Die relevanten Informationen zur Todesursachenstatistik entstammen der Todesbescheinigung, auf der der leichenschauende Arzt die medizinischen Ursachen für den Tod vermerkt. Der Ablauf gestaltet sich wie folgt: Im Falle eines Todes muss die Person von einem Arzt untersucht und für tot erklärt werden. Dabei erfasst er in der Todesbescheinigung unter anderem diejenigen Ursachen, die verantwortlich für den Tod waren, sowie Begleiterkrankungen. Anschließend geht die Todesbescheinigung an das Standesamt des Sterbeortes, um fehlende demografische Angaben zu ergänzen. Der vertrauliche Teil mit den Angaben zu den Todesursachen wird im nächsten Schritt an das Gesundheitsamt versendet, wo unter anderem die Informationen auf Plausibilität und Vollständigkeit geprüft werden sollen. Nach Prüfung der Angaben geht die Todesbescheinigung an das zuständige statistische Landesamt, wo die Daten im Rahmen der Todesursachenstatistik aufbereitet werden. Parallel dazu wird die im Standesamt erstellte Sterbefallzählkarte mit den demografischen Angaben der Person direkt an das statistische Landesamt geschickt.

Die Aufbereitung der Daten für die Todesursachenstatistik geschieht nach dem weltweit gültigen Regelwerk der WHO und beinhaltet die Auswahl des Grundleidens und seine Umschlüsselung in ICD-Codes entweder manuell oder elektronisch. Nach Aggregation der Landesergebnisse werden diese an das Statistische Bundesamt gesandt, das aus den Länderergebnissen ein Bundesergebnis macht und dieses veröffentlicht. Damit handelt es sich bei der Todesursachenstatistik um eine klassische dezentrale Statistik, zudem basiert sie auf einer Vollerhebung, da jeder Todesfall erfasst wird. Aufgrund dessen wird auf die Darstellung von Konfidenzintervallen und Signifikanztests verzichtet.

Die Ergebnisse werden in der Regel im August nach Abschluss des Berichtsjahres veröffentlicht, wobei der Schwerpunkt auf der deskriptiven Darstellung der Ergebnisse der Todesursachenstatistik liegt.

#### Exkurs: Todesbescheinigung

Die Regelungen zur Leichenschau und damit auch zur Todesbescheinigung obliegen aufgrund der Länderkompetenz den einzelnen Ländern. Dies hat zur Folge, dass jedes Bundesland auf der Grundlage des Bevölkerungsstatistikgesetzes eigene Regelungen für die Leichenschau erlassen hat, die in einigen Punkten voneinander abweichen.

Der leichenschauende Arzt vermerkt auf der Todesbescheinigung unter anderem die zum Tode führenden Ursachen. Dies geschieht papierbasiert und handschriftlich als Klartext, nicht in Form von ICD-Codes. Die dokumentierten Todesursachen sind alle Krankheiten oder Verletzungen, die den Tod verursacht haben oder dazu beigetragen haben, einschließlich anderer Diagnosen, die aus Sicht des Arztes relevant sind. Ergänzt werden die Angaben durch die Nennung von Zeitabständen zwischen dem Beginn des jeweiligen Krankheitszustandes und dem Todeseintritt und die Umstände des Todes (bspw. Unfall). Die Definition der zum Tode führenden Krankheiten soll sicherstellen, dass nur bedeutende Angaben auf der Todesbescheinigung stehen, triviale Diagnosen wie Herz-Kreislauf-Versagen oder Atemstillstand sollen nicht Gegenstand der Todesbescheinigung sein.

Abbildung Z1 (siehe Onlinematerial) zeigt den Vordruck der WHO zur Todesbescheinigung. Dieser ist als Mindestanforderung zu sehen und kann erweitert und angepasst werden. Wichtig ist, dass die Todesbescheinigung keinen Raum für unzusammenhängende Texte gibt. Elementarer Bestandteil ist daher die Struktur, anhand welcher der leichenschauende Arzt die Todesursachen einträgt und die eine Kausalkette von der Krankheit, die initial ursächlich den Tod ausgelöst hat, bis hin zur unmittelbaren Todesursache darstellt. In Teil 1 werden oben die unmittelbaren Todesursachen vermerkt und weiter darunter diejenigen Ursachen, welche die unmittelbaren Ursachen bedingt haben (Kausalkette). In Zeile 1.d wird die initiale Ursache eingetragen, die ursächlich für den Tod war und alle anderen nachfolgenden Ursachen bedingt hat. Hierbei handelt es sich um das sogenannte Grundleiden. Das Grundleiden ist jene Erkrankung, die den Sterbeprozess in Gang gesetzt hat. Daher kommt ihm eine besondere Bedeutung zu, da aktuell nur diese Ursache in die Todesursachenstatistik einfließt (unikausale Todesursachenstatistik).

Die Qualität der Todesursachenstatistik steht und fällt damit mit den Informationen, die der leichenschauende Arzt auf der Todesbescheinigung dokumentiert.

Der Suizid ist insofern eine Besonderheit, da er in einigen Fällen nicht leicht zu erkennen ist. Ein Sprung von einem Gebäude mit vorherigem Abschiedsbrief ist einem Suizid leicht zuzuordnen, ein Suizid mittels Tabletten hingegen ist nicht immer direkt ersichtlich. Erschwerend kommt hinzu, dass gerade bei Personen höheren Alters ein Suizid nicht direkt in den Fokus des untersuchenden Arztes kommt, da es sich dabei aufgrund des hohen Alters durchaus um „erwartete“ Todesfälle handeln kann. Daher sind die hier dargestellten Ergebnisse als Untergrenze der tatsächlichen Zahl der Suizide zu sehen.

## Ergebnisse der Todesursachenstatistik

### Allgemeiner Überblick

Im Jahr 2019 starben in Deutschland insgesamt 939.520 Personen, davon 9041 Personen durch Suizid. Das entspricht einem Anteil von knapp 1 %. Im langfristigen Vergleich mit den Ergebnissen des Berichtsjahres 1980 ist bei der Zahl der Suizide ein deutlicher Rückgang um 51,0 % zu verzeichnen (1980: 18.451 Verstorbene durch Suizid; Tab. 1). Auch im mittelfristigen Vergleich zeigt sich, dass ein Rückgang weiterhin anhält: So waren es im Jahr 1998 noch insgesamt 11.644 Personen, die ihrem Leben vorzeitig ein Ende gesetzt haben. Dieser grundsätzliche Rückgang im Vergleich zu den Ergebnissen von 1980 und 1998 ist zwar als positiv zu bewerten, diese Entwicklungen unterliegen jedoch jährlichen, teils zufallsbedingten Schwankungen: So schwankt die Veränderungsrate der absoluten Anzahl der Suizide verglichen zum Vorjahr von −7,4 % (Vergleich 1986 zu 1985) bis hin zu +4,2 % (Vergleich 2010 zu 2009).

**Table 1:** 

Jahr	Anzahl der Suizide			Anteile an insgesamt	
	Insgesamt	Männer	Frauen	Männer [%]	Frauen [%]
1980	18.451	11.789	6662	63,9	36,1
1981	18.825	12.192	6633	64,8	35,2
1982	18.711	12.274	6437	65,6	34,4
1983	18.417	11.921	6496	64,7	35,3
1984	17.677	11.610	6067	65,7	34,3
1985	17.571	11.797	5774	67,1	32,9
1986	16.269	10.792	5477	66,3	33,7
1987	16.625	11.124	5501	66,9	33,1
1988	15.590	10.527	5063	67,5	32,5
1989	14.567	9922	4645	68,1	31,9
1990	13.924	9534	4390	68,5	31,5
1991	14.011	9656	4355	68,9	31,1
1992	13.458	9326	4132	69,3	30,7
1993	12.690	8960	3730	70,6	29,4
1994	12.718	9130	3588	71,8	28,2
1995	12.888	9222	3666	71,6	28,4
1996	12.225	8728	3497	71,4	28,6
1997	12.265	8841	3424	72,1	27,9
1998	11.644	8575	3069	73,6	26,4
1999	11.157	8080	3077	72,4	27,6
2000	11.065	8131	2934	73,5	26,5
2001	11156	8188	2968	73,4	26,6
2002	11.163	8106	3057	72,6	27,4
2003	11.150	8179	2971	73,4	26,6
2004	10.733	7939	2794	74,0	26,0
2005	10.260	7523	2737	73,3	26,7
2006	9765	7225	2540	74,0	26,0
2007	9402	7009	2393	74,5	25,5
2008	9451	7039	2412	74,5	25,5
2009	9616	7228	2388	75,2	24,8
2010	10.021	7465	2556	74,5	25,5
2011	10.144	7646	2498	75,4	24,6
2012	9890	7287	2603	73,7	26,3
2013	10.076	7449	2627	73,9	26,1
2014	10.209	7624	2585	74,7	25,3
2015	10.078	7397	2681	73,4	26,6
2016	9838	7374	2464	75,0	25,0
2017	9235	6985	2250	75,6	24,4
2018	9396	7111	2285	75,7	24,3
2019	9041	6842	2199	75,7	24,3

Unter den insgesamt 9041 durch Suizid verstorbenen Personen im Jahr 2019 waren 6842 Männer und 2199 Frauen – der Suizid ist mit einem Anteil von 75,7 % deutlich männerdominiert. Zum Vergleich: Der Anteil der Männer und Frauen in der Gesamtbevölkerung ist fast gleich und lag im Jahr 2019 bei 49,3 % Männern (41.037.613) und 50,7 % Frauen (42.129.098). Damit war Suizid für ca. jeden 68. Sterbefall bei Männern und ca. jeden 215. Sterbefall bei Frauen die Todesursache.

Der Anteil der Männer an den Suiziden war nicht immer so hoch: Verglichen mit den Daten ab 1980 zeigt sich, dass dieser Anteil stetig angewachsen ist, von ehemals 63,9 % auf nun 75,7 %. Darüber hinaus zeigt sich im langfristigen Vergleich der Jahre 1980 und 2019 auch, dass der Rückgang der Suizidzahlen bei Frauen stärker (−67,0 %) als bei den Männern (−42,0 %) ausgefallen ist.

Die Abbildung absoluter Zahlen gibt zunächst einen Überblick über das Ausmaß der Fälle. So ist die Aussage, dass über 9000 Todesfälle auf einen Suizid zurückzuführen sind, für viele Leser besser greifbar als eine Aussage anhand von Sterbeziffern (Zahl der Sterbefälle bezogen auf die Bevölkerung). Die absoluten Zahlen sagen jedoch nichts über das Risiko aus, an bestimmten Ursachen zu versterben. Ein Sterberisiko hängt immer auch mit der Bevölkerungsstruktur zusammen. Je älter die Bevölkerung ist, desto mehr Todesfälle durch Krebs gibt es, ohne dass sich das Risiko, daran zu versterben, erhöht hat. An dieser Stelle hilft die Altersstandardisierung der Sterbeziffern, mit deren Hilfe die Einflüsse durch sich ändernde demografische Rahmenbedingungen beseitigt werden. Erst nach Altersstandardisierung der Sterbeziffern können bevölkerungsunabhängige Vergleiche auch zwischen strukturell unterschiedlichen Bevölkerungsgruppen gemacht werden [4]. Dadurch ist eine Betrachtung von Todesursachen und Krankheiten aus epidemiologischer Sicht möglich, ohne dass die Ergebnisse bspw. durch Veränderungen in der Bevölkerungsstruktur verfälscht werden.

Bei der Betrachtung der altersstandardisierten Sterbeziffer zeigt sich folgendes Bild: Verglichen mit den Ergebnissen der Vorjahre liegt die altersstandardisierte Sterberate mit 10,6 Sterbefällen je 100.000 Einwohner im Jahr 2019 auf einem historisch niedrigen Niveau (Tab. 2). Im Vergleich der Jahre 1998 bis 2019 ist das der niedrigste Wert; der mit 15,4 Sterbefällen je 100.000 Einwohner höchste Wert in dieser zeitlichen Betrachtung stammt aus dem Jahr 1998. Die Entwicklung ist zwar grundsätzlich positiv in der Hinsicht, dass insgesamt die Zahlen gesunken sind, jedoch zeigt sich, wie auch bei den absoluten Zahlen, dass diese Entwicklung jährlichen Schwankungen unterliegt. In den Jahren 2010 bis 2019 zeichnet sich zudem eine leichte Stagnation ab.

**Table 2:** 

Jahr	Insgesamt	Männer	Frauen
1998	15,4	24,0	8,0
1999	14,7	22,4	7,9
2000	14,5	22,5	7,6
2001	14,5	22,3	7,6
2002	14,5	22,0	7,8
2003	14,4	22,0	7,5
2004	13,7	21,1	7,0
2005	13,0	19,9	6,8
2006	12,3	18,8	6,3
2007	11,8	18,1	5,9
2008	11,7	17,9	5,9
2009	11,9	18,3	5,8
2010	12,3	18,7	6,2
2011	12,6	19,5	6,1
2012	12,2	18,4	6,3
2013	12,4	18,6	6,3
2014	12,4	18,8	6,2
2015	12,1	17,9	6,4
2016	11,7	17,6	5,8
2017	10,9	16,5	5,3
2018	11,0	16,6	5,3
2019	10,6	15,9	5,1

Diese Entwicklung lässt sich sowohl insgesamt als auch bei Männern und Frauen feststellen. Deutliche Unterschiede gibt es wie auch bei den absoluten Zahlen zwischen den Geschlechtern: Das Sterberisiko der Männer lag in 2019 mit 15,9 Fällen je 100.000 Einwohner über dreimal so hoch wie bei den Frauen mit 5,1 Sterbefällen je 100.000 Einwohner.

### Vorläufige Ergebnisse für das Jahr 2020

Die Todesursachenstatistik wurde in der Vergangenheit immer als Jahresstatistik aufbereitet und veröffentlicht, Ergebnisse liegen in der Regel Mitte August nach Abschluss des Berichtsjahres vor. Die Coronapandemie hat noch einmal einen viel stärkeren Fokus auf die Ergebnisse der amtlichen Gesundheitsstatistiken und damit auch auf die Todesursachenstatistik gelegt. Um dem gestiegenen Bedarf nach aktuelleren Daten gerecht zu werden, werden ab Juli 2021 beginnend mit dem Berichtsmonat Januar 2020 monatliche Daten zur Todesursachenstatistik aufbereitet und veröffentlicht. Die monatlichen Berichte in der Todesursachenstatistik stellen vorläufige Daten dar, bilden den jeweiligen Bearbeitungsstand zum monatlichen Stichtag ab und können sich durch Nachmeldungen oder Korrekturen noch verändern. Das bedeutet, dass die Qualität der Berichte mit zunehmendem Vollständigkeitsgrad steigt. Die monatlichen Ergebnisse werden dabei im Rahmen eines deutlich reduzierten Merkmalskranzes ausgewiesen, in dem die Hauptkapitel der ICD-10, wichtige Todesursachengruppen und einige relevante Einzeldiagnosen enthalten sind.

Aufgrund des gewachsenen Interesses an den Daten zu Suiziden in Pandemiezeiten wurde dieser Aspekt im Merkmalskranz bei der Aufbereitung der monatlichen Berichte berücksichtigt.

Im Jahr 2020 wurden bislang (Stand 20.09.2021) insgesamt 9237 Todesfälle durch Suizid gezählt. Die meisten Suizide absolut gab es demnach in den Monaten Juni (844) und Mai (838; Tab. 3). Berücksichtigt man die unterschiedliche Anzahl an Tagen je Monat, stellt sich eine etwas andere Situation dar: Zwar ist nach wie vor der Monat Juni der mit den meisten Suiziden (28 Suizide/Tag), an zweiter Stelle liegt nun jedoch der Monat Februar mit 27,5 Suiziden pro Tag. Das Vorjahresniveau von 9041 Suiziden wurde entgegen mancher Vermutungen damit nur leicht überstiegen.

**Table 3:** 

Monat	Anzahl der Suizide
Januar	761
Februar	771
März	772
April	736
Mai	838
Juni	844
Juli	810
August	796
September	754
Oktober	754
November	730
Dezember	671
Insgesamt	9237

### Suizide nach Sterbealter

Im Jahr 2019 betrug das durchschnittliche Alter der Personen, die ihrem Leben vorzeitig ein Ende gesetzt haben, 58,5 Jahre. Die Unterschiede zwischen Frauen und Männern waren gering, während die Männer im Schnitt 58,2 Jahre alt waren, lag das durchschnittliche Sterbealter bei den Frauen bei 59,7 Jahren. In der Entwicklung von 1998 bis 2019 zeigt sich, dass das durchschnittliche Sterbealter zwar nur langsam, aber recht konstant über die Zeit hinweg zugenommen hat: So lag das durchschnittliche Sterbealter im Jahr 1998 bei insgesamt 53,2 Jahren, bei Männern bei 51,6 und bei Frauen bei 57,6 Jahren.

Verglichen mit dem allgemeinen Sterbealter versterben diejenigen, die aufgrund von Suizid aus dem Leben scheiden, sehr jung: Das durchschnittliche Sterbealter über alle Fälle hinweg liegt mit 78,5 Jahren um genau 20 Jahre über dem Sterbealter bei Suizid. Die Männer versterben 17,4 Jahre früher als der Durchschnitt, die Frauen 21,7 Jahre.

Relativ betrachtet ist damit das durchschnittliche Sterbealter bei Suizid stärker angestiegen als das durchschnittliche Sterbealter insgesamt: Während von 1998 bis 2019 das Sterbealter bei Suizid um 10 % zugenommen hat (+5,3 Jahre), ist es bei allen Sterbefällen lediglich um 4,4 % (+3,3 Jahre) angestiegen. Allerdings betrifft diese Entwicklung vor allem die Männer: Das Sterbealter bei den Männern aufgrund Suizids erhöhte sich im gleichen Zeitraum um 12,8 % oder 6,6 Jahre und bei den Frauen nur um 3,6 % oder 2,1 Jahre.

### Suizide nach Altersgruppen

Suizide finden nahezu über alle Altersgruppen hinweg statt (Tab. 4). Ab welchem Alter eine Person bewusst Suizid begeht oder begehen kann, ist nicht leicht zu bestimmen. Es werden keine Fälle von Suiziden in der Altersgruppe bis unter 10 Jahren registriert, vereinzelt und selten finden Suizide allerdings bereits in der Altersgruppe der 10- bis unter 15-Jährigen statt. Aus diesem Grund werden die unteren Altersgruppen der 0‑ bis 15-Jährigen bei der Betrachtung der Suizide zusammengefasst, alle anderen werden in 5‑Jahres-Altersklassen ausgewiesen und nur bei den über 90-Jährigen aufgrund der geringen Fallzahl wieder aggregiert. Die absoluten Zahlen steigen mit dem Alter an und liegen in den Altersgruppen zwischen 50 und 60 Jahren am höchsten, vor allem bei den Männern: Von den 2667 im Jahr 2019 an Suizid Verstorbenen 50- bis 65-Jährigen waren 1974 Männer (74 %) und 693 Frauen (26 %). Dies liegt vor allem daran, dass es in diesen Altersgruppen sehr viele Menschen gibt. Die Suizidrate je 100.000 Einwohner steigt mit dem Alter an und liegt bei den 85- bis 90-Jährigen am höchsten.

**Table 4:** 

Altersgruppen	Anzahl		
	Insgesamt	Männer	Frauen
< 15 Jahre	22	11	11
15 bis < 20 Jahre	163	121	42
20 bis < 25 Jahre	286	235	51
25 bis < 30 Jahre	361	289	72
30 bis < 35 Jahre	417	329	88
35 bis < 40 Jahre	449	367	82
40 bis < 45 Jahre	481	381	100
45 bis < 50 Jahre	599	451	148
50 bis < 55 Jahre	924	667	257
55 bis < 60 Jahre	931	707	224
60 bis < 65 Jahre	812	600	212
65 bis < 70 Jahre	653	490	163
70 bis < 75 Jahre	573	413	160
75 bis < 80 Jahre	849	649	200
80 bis < 85 Jahre	775	578	197
85 bis < 90 Jahre	518	400	118
90 Jahre und älter	228	154	74
Alle Altersgruppen	9041	6842	2199

**Figure 1:**
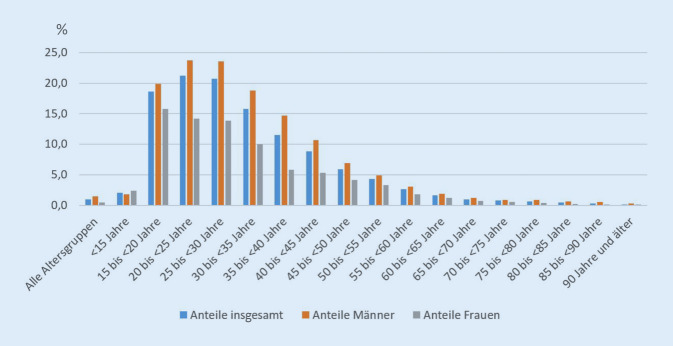

Ein anderes Bild ergibt sich, wenn man die Bedeutung des Suizids unter allen Todesursachen in der jeweiligen Altersgruppe betrachtet: Hier zeigt sich, dass sowohl in der Altersgruppe der 20- bis 25-Jährigen als auch der 25- bis 30-Jährigen der Anteil an allen Todesursachen über 20 % liegt (Abb. 1). Hierbei gibt es deutliche Unterschiede zwischen den Geschlechtern: Der Anteil der Suizide bei den Männern in den gerade genannten Altersgruppen beträgt 23,7 % und 23,6 %, bei den Frauen 14,2 % und 13,9 %. Dieser Unterschied ist in allen weiteren Altersgruppen zu finden, lediglich bei den unter 15-Jährigen ist der Anteil bei den Mädchen größer (2,4 % weiblich zu 1,8 % männlich).

### Suizide nach Region

Das Niveau der suizidalen Sterblichkeit weist im Vergleich der Bundesländer deutliche Unterschiede auf. In Deutschland betrug die altersstandardisierte Sterbeziffer bei Suizid im Jahr 2019 insgesamt 10,6 Fälle je 100.000 Einwohner (Tab. 5). Die geringsten Werte sind in Nordrhein-Westfalen (7,4), Bremen (8,9) und Brandenburg (10,1) zu finden. Die höchsten Werte finden sich in Sachsen-Anhalt (13,5), Sachsen (13,0) und Hamburg (12,7) wieder. Auch in der zeitlichen Entwicklung gibt es Unterschiede. In Gesamtdeutschland ist die altersstandardisierte Sterblichkeit aufgrund von Suizid von 1998 bis 2019 um 31,2 % gesunken. Diese positive Entwicklung ist nicht überall im gleichen Maße zu finden: Die größten Rückgänge sind in Bremen (−47,0 %), Brandenburg (−43,3 %) und Thüringen (−39,5 %) zu finden, die geringsten in Mecklenburg-Vorpommern (−7,0 %) und Hessen (−13,0 %). Im Saarland war seit 1998 sogar eine leichte Zunahme um 18,8 % zu verzeichnen. Dies ist allerdings auch durch einen sehr niedrigen Ausgangswert im Jahr 1998 bedingt.

**Table 5:** 

Region	Altersstandardisierte Sterbeziffer		Entwicklung 2019 zu 1998 In %
	1998	2019	
*Deutschland*	*15,4*	*10,6*	*−31,2*
Baden-Württemberg	16,4	11,0	−32,9
Bayern	17,5	11,4	−34,9
Berlin	13,9	10,4	−25,2
Brandenburg	17,8	10,1	−43,3
Bremen	16,8	8,9	−47,0
Hamburg	20,4	12,7	−37,7
Hessen	13,1	11,4	−13,0
Mecklenburg-Vorpommern	12,9	12,0	−7,0
Niedersachsen	15,3	10,5	−31,4
Nordrhein-Westfalen	11,4	7,4	−35,1
Rheinland-Pfalz	16,7	11,7	−29,9
Saarland	9,6	11,4	18,8
Sachsen	20,3	13,0	−36,0
Sachsen-Anhalt	19,2	13,5	−29,7
Schleswig-Holstein	16,7	12,6	−24,6
Thüringen	19,5	11,8	−39,5

^a^Anhand der Standardbevölkerung „Deutschland 2011“

### Suizidmethoden

Die wissenschaftliche Literatur zum Thema Suizid unterscheidet die Methoden unterschiedlich. So wird nach einer Klassifikation von Bochnik [5] zwischen „weichen“ und „harten“ Methoden unterschieden. Zu den weichen Methoden werden dabei die tödliche Einnahme von Tabletten oder Drogen im weitesten Sinne sowie Vergiftungen (einschließlich Tod durch Einatmen von Abgasen usw.) gezählt. Zu den harten Suizidmethoden zählen demnach das Erhängen, Erschießen, Ertrinken, Sturz aus der Höhe oder vor einen sich bewegenden Gegenstand und tiefe Schnitte. Andere Typologien differenzieren zwischen aktiven Methoden (Schießen, Sturz, Ertränken, Erhängen, Schnitte) und passiven Methoden (Tabletten, Gifte und Gase). Eine Unterscheidung der Suizidmethoden ist insofern relevant, da der Gesetzgeber Einfluss auf die Suizidwege nehmen kann, indem z. B. bei entsprechenden Medikamenten die Verpackungsgröße minimiert, der Zugang zu bestimmten Mitteln wie Waffen etc. beschränkt und der Zugang zu hohen Gebäuden und/oder Brücken erschwert wird.

Als Suizidmethoden werden in allen Altersgruppen überwiegend sogenannte harte Methoden wie Erhängen, Erdrosseln und Ersticken (ICD-10: X70) angewandt (siehe Onlinematerial, Tabelle Z1). Sie machten im Jahr 2019 fast die Hälfte aller Suizidmethoden aus (45,1 %), das waren insgesamt 4074 Personen (3358 Männer und 716 Frauen). Insbesondere bei den Männern wurden harte Methoden häufiger angewendet: Während 49,1 % der durch Suizid verstorbenen Männer Erhängen, Erdrosseln oder Ersticken als Suizidmethode wählten, waren es nur 32,6 % der Frauen. Unabhängig vom Alter stellten die „Vorsätzlichen Selbstvergiftungen“ (ICD-10: X60–X69) mit 1550 Fällen die zweithäufigste Suizidmethode dar (936 Männer, 614 Frauen). Im Vergleich zu den Frauen mit 27,9 % lag der Anteil der Männer mit dieser Methode bei lediglich 13,7 %. Den größten Anteil bei den „Vorsätzlichen Selbstvergiftungen“ hatten sowohl bei den Männern wie auch bei den Frauen insbesondere die „Vorsätzliche Selbstvergiftung durch und Exposition gegenüber sonstige(n) und nicht näher bezeichnete(n) Arzneimittel(n), Drogen und biologisch aktive(n) Substanzen“ (ICD-10: X64). Für 13,5 % aller Frauen und 6,9 % aller Männer war dies die gewählte Art des Suizids.

### Suizide nach Sterbemonaten

Die Todesursachenstatistik war lange Zeit ausschließlich als Jahresstatistik konzipiert. Mit der Umstellung der Erhebungsmodalitäten besteht aber auch die Möglichkeit, die Daten nach dem Sterbemonat auszuwerten, um einen Überblick über das suizidale Verhalten im Laufe eines Jahres zu erhalten. So lassen sich beispielsweise Fragen untersuchen wie: „Starben im Zeitraum von sportlichen Großereignissen mehr Menschen am Herzinfarkt?“ und: „Stimmt es, dass mehr Menschen in der dunklen Jahreszeit/Wintermonaten ihrem Leben ein Ende setzen als in den übrigen Monaten des Jahres?“ Da die Todesursachenstatistik die Sterbefälle nach dem sogenannten Ereignisdatum (= Sterbedatum) ausweist, ist der Sterbetag eindeutig dem jeweiligen Monat zuzuordnen.

Für das Berichtsjahr 2019 kann die allgemein geläufige Annahme, dass sich in den Wintermonaten mehr Menschen das Leben nehmen, nicht bestätigt werden (Tab. 6). Grundsätzlich zeigen die monatlichen Ergebnisse, dass es eine gleichmäßige Verteilung über das gesamte Jahr gibt. Die Monate mit den höchsten Anteilen sind der Juli (8,9 % Anteil am Jahresergebnis) und der April (8,7 %). Die geringsten Werte sind in den Monaten September (7,3 % Anteil am Jahresergebnis) und Februar (7,8 %) feststellbar.

**Table 6:** 

Sterbemonat	Insgesamt	Anteil am Jahresergebnis in %
Januar	776	8,6
Februar	704	7,8
März	765	8,5
April	786	8,7
Mai	767	8,5
Juni	768	8,5
Juli	808	8,9
August	778	8,6
September	660	7,3
Oktober	760	8,4
November	756	8,4
Dezember	713	7,9
Insgesamt	9041	100

**Figure 2:**
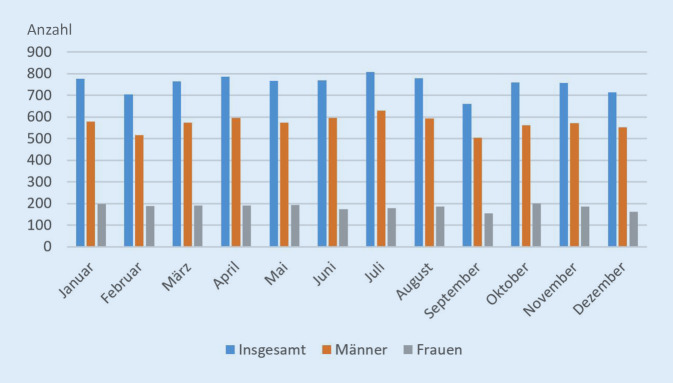

Auch bei der differenzierten Darstellung der Monatsergebnisse nach Geschlecht ergibt sich kaum eine Besonderheit: Zum einen sind Männer grundsätzlich aufgrund der höheren Zahlen überrepräsentiert, zum anderen zeigen sich über das Jahr hinweg nur geringe Schwankungen (Abb. 2).

## Fazit und Ausblick

Bei fast 1 % aller Gestorbenen im Jahr 2019 war der Suizid die Todesursache. Dies zeigen die Ergebnisse der Todesursachenstatistik, in die alle Daten zu den Gestorbenen mit Hauptwohnsitz in Deutschland einfließen und die neben den eigentlichen Todesursachen auch Informationen zu Alter und Geschlecht beinhalten. Dabei ist deutlich geworden, dass das Merkmal Alter beim Thema Suizid eine wichtige Rolle spielt: Absolut gesehen werden die meisten Suizide von Menschen der Altersgruppe 50 bis 65 Jahre begangen, auf 100.000 Einwohner gerechnet jedoch ist die Suizidrate bei den 85- bis 90-Jährigen am höchsten.

Aktuell werden die Daten der Todesursachenstatistik immer noch unikausal aufbereitet, das bedeutet, dass von allen auf der Todesbescheinigung vorhandenen Informationen ausschließlich das sogenannte Grundleiden ausgewiesen wird. Langfristig aber steigt zu Recht die Erwartung, alle vorliegenden Informationen auszuwerten, um bspw. Rückschlüsse auf mögliche Risikofaktoren aufzuzeigen und frühzeitig auf diese im Rahmen geeigneter Präventionsmaßnahmen reagieren zu können. Bei Suiziden liegen oftmals psychische Begleiterkrankungen wie Depressionen zugrunde. Die gestiegene Relevanz der Komorbiditäten trifft aber nicht nur beim Thema Suizid zu: Aufgrund der immer höher werdenden Lebenserwartung sterben die Menschen heute nicht wie vor 200 Jahren im mittleren Alter an nur einer Krankheit, sondern deutlich älter und an einer Vielzahl von Krankheiten (Multimorbidität). Es ist wichtig, die Wechselwirkungen zwischen verschiedenen Krankheiten abzubilden, um diese bekämpfen zu können. Im Bereich Statistik wird diese Schwachstelle bereits angegangen, so steht den statistischen Landesämtern ein elektronisches System [6] zur automatisierten Codierung der Todesursachen zur Verfügung (Iris/MUSE), das weltweit als Standard gilt und eine multikausale Aufbereitung aller auf der Todesbescheinigung vorhandenen Informationen ermöglicht und auswertbar macht. Erste Ergebnisse liegen voraussichtlich Ende 2022 vor. Ein weiterer Schritt in die Zukunft wäre die Bereitstellung einer webbasierten Plattform zur direkten Erfassung der Todesursachen vor Ort, bspw. mittels einer App. Auch diese Möglichkeit wird aktuell auf Machbarkeit geprüft. Im Ergebnis würde die Qualität der Eingaben deutlich erhöht werden können, wenn unplausible Angaben im Dialog zwischen App und Arzt geklärt würden. Außerdem lägen dann alle Informationen direkt in elektronischer Form vor und könnten automatisiert verarbeitet, weitergeleitet und sofort verfügbar gemacht werden. Um dies alles effektiv und reibungsfrei entwickeln zu können, wäre es wünschenswert, wenn in Deutschland eine bundeseinheitliche Todesbescheinigung verwendet wird, und nicht – wie aktuell – 16 verschiedene [7].

Parallel zu diesen Verbesserungen ist auch eine qualitativ bessere Leichenschau wünschenswert, ähnlich dem Coroner-System^2^ in England. Das Ausfüllen der Todesbescheinigung sollte seitens der Ärzte nicht bloß als Formalität, sondern als Quelle von Informationen mit hoher Relevanz für die erfolgreiche Ermittlung der zum Tode führenden Krankheiten angesehen werden. Darüber hinaus wäre auch eine Erhöhung des Anteils an Obduktionen wünschenswert, da nur darüber zuverlässig detaillierte Ergebnisse zu den Todesursachen erzielt werden können.

Wie auch immer eine künftige Todesursachenstatistik aussehen und die Verarbeitung der Daten im Zeitalter der Digitalisierung verbessert werden wird: Nach wie vor sterben jedes Jahr fast 10.000 Menschen in Deutschland durch Suizid, das sind über 27 Personen täglich. Jeder einzelne dieser Fälle stellt eine Tragödie dar und sollte von der Gesellschaft nicht einfach hingenommen werden.

## Supplementary Information





## Footnotes

1 Zuletzt geändert durch Artikel 3 des Gesetzes vom 09.06.2021 (Bundesgesetzblatt Teil I S. 1649).

2 Im Gegensatz zu Deutschland, wo jeder Arzt eine Todesbescheinigung ausstellen kann/muss, wird diese Tätigkeit in England von speziell dafür geschulten Personen, sogenannten Coroner, durchgeführt. Coroner sind in der Regel Ärzte, die über weitergehendes Wissen zu rechtlichen und klinischen Fragen rund um die Todesbescheinigungen verfügen.
