# Supplementary material for: Suicides in Germany: results from the official cause of death statistics
Source: Bundesgesundheitsblatt Gesundheitsforschung Gesundheitsschutz. 2021 Dec 28;65(1):3–10. [Article in German] doi: 10.1007/s00103-021-03470-2 (PMC8732928; doi:10.1007/s00103-021-03470-2)
Supplement: Supplementary file 1 [file 103_2021_3470_MOESM1_ESM.pdf]

Bundesgesundheitsblatt – Gesundheitsforschung – Gesundheitsschutz, Ausgabe 1/2022

Onlinematerial zum Beitrag:

Suizide in Deutschland: Ergebnisse der amtlichen Todesursachenstatistik

Torsten Schelhase[1]

[1] Statistisches Bundesamt, Zweigstelle Bonn, H11 Gesundheitsstatistiken, Bonn, Deutschland

Korrespondenzadresse:

Torsten Schelhase

Statistisches Bundesamt, Zweigstelle Bonn

Referatsleiter H11 Gesundheitsstatistiken

Graurheindorfer Straße 198

53117 Bonn

torsten.schelhase@destatis.de

Inhalt:

Abbildung Z1: Internationales Formular der Todesursachenbescheinigung der Weltgesundheitsorganisation (WHO)

Tabelle Z1: Methoden des Suizids absolut und Anteil an allen Suiziden nach Geschlecht in Deutschland, 2019

Abbildung Z1: Internationales Formular der Todesursachenbescheinigung der Weltgesundheitsorganisation (WHO)

**Angaben zur Person** (hier sind weitere Angaben je nach Erfordernis des Landes möglich)			
Geschlecht	☐ Weiblich	☐ Männlich	☐ Nicht bekannt
Geburtsdatum	T T M M J J J J	Sterbedatum	T T M M J J J J

**Abschnitt 1 - Medizinische Angaben: Teil 1 und 2**			
1. Direkt zum Tode führende Krankheit oder Zustand in Zeile a eintragen<br>Krankheiten oder Zustände als Kausalkette eintragen (wenn zutreffend)<br>Zugrunde liegende Krankheit oder Zustand auf der untersten benutzten Zeile eintragen		Todesursache	Zeitintervall vom Beginn bis zum Tod
	a		
	b	Durch:	
	c	Durch:	
	d	Durch:	
2. Weitere wesentliche Krankheiten oder Zustände, die zum Tode beigetragen haben (Zeitintervall kann in Klammern hinter den Zustand geschrieben werden)			

**Abschnitt 2 - Weitere medizinische Angaben**			
Wurde innerhalb der letzten 4 Wochen eine Operation durchgeführt?	☐ Ja	☐ Nein	☐ Nicht bekannt
Falls Ja, geben Sie das Datum der Operation an	T T M M J J J J		
Falls Ja, geben Sie den Grund für die Operation an (Krankheit oder Zustand)			
Wurde eine Autopsie beantragt?	☐ Ja	☐ Nein	☐ Nicht bekannt
Wenn Ja, wurden die Ergebnisse in diesem Schein bereits berücksichtigt?	☐ Ja	☐ Nein	☐ Nicht bekannt

**Todesart**		
☐ Krankheit	☐ Tätlicher Angriff	☐ Konnte nicht ermittelt werden
☐ Unfall	☐ Gesetzliche Maßnahme	☐ Offene Ermittlung
☐ Suizid	☐ Krieg	☐ Unbekannt
Falls eine äußere Ursache oder Vergiftung vorliegt:	Datum der äußeren Ursache oder Vergiftung	T T M M J J J J
Beschreiben Sie, wie es zur äußeren Ursache kam (bei Vergiftung die Substanz angeben)		

**Ort des Ereignis der äußeren Ursache** (wenn zutreffend)			
☐ Zuhause	☐ Wohnheim oder -anstalt	☐ Schule, sonstige öffentliche Gebäude	☐ Sportstätte
☐ Straße oder Weg	☐ Gewerbe- oder Dienstleistungseinrichtung	☐ Industrieanlage oder Baustelle	☐ Landwirtschaftlicher Betrieb
☐ Sonstiger näher bezeichneter Ort:			☐ Nicht bekannt

**Fetal- oder Säuglingssterbefall** (wenn zutreffend)			
Mehrlingsschwangerschaft	☐ Ja	☐ Nein	☐ Nicht bekannt
Totgeburt	☐ Ja	☐ Nein	☐ Nicht bekannt
Bei Tod innerhalb von 24 Stunden: gelebte Stunden		Geburtsgewicht (in Gramm)	
Vollendete Wochen der Schwangerschaft		Alter der Mutter (in Jahren)	
Falls es sich um einen Perinatalsterbefall handelt, dokumentieren Sie die Krankheiten der Mutter, die Auswirkungen auf den Fetus und das Neugeborene hatten			

**Lag eine Schwangerschaft vor?**	☐ Ja	☐ Nein	☐ Nicht bekannt
☐ Zum Zeitpunkt des Todes	☐ Innerhalb von 42 Tagen vor dem Tod		
☐ Zwischen 43 Tage und bis zu einem Jahr vor dem Tod	☐ Nicht bekannt		
Hat die Schwangerschaft zum Tode beigetragen?	☐ Ja	☐ Nein	☐ Nicht bekannt

Tabelle Z1: Methoden des Suizids absolut und Anteil an allen Suiziden nach Geschlecht in Deutschland, 2019

Methoden mit ICD-10-Code	Anzahl absolut			Anteil an allen Suiziden		
	Insgesamt	Männer	Frauen	Insgesamt	Männer	Frauen
X60 Vorsätzliche Selbstvergiftung durch und Exposition gegenüber nicht opioidhaltige(n) Analgetika, Antipyretika und Antirheumatika	8	3	5	0,1%	0,0%	0,2%
X61 Vorsätzliche Selbstvergiftung durch und Exposition gegenüber Antiepileptika, Hypnotika, Antiparkinsonmittel(n) und psychotrope(n) Substanzen, anderenorts nicht klassifiziert	217	99	118	2,4%	1,4%	5,4%
X62 Vorsätzliche Selbstvergiftung durch und Exposition gegenüber Betäubungsmittel und Psychodysleptika [Halluzinogene], anderenorts nicht klassifiziert	105	55	50	1,2%	0,8%	2,3%
X63 Vorsätzliche Selbstvergiftung durch und Exposition gegenüber sonstige(n) Arzneimittel(n) mit Wirkung auf das autonome Nervensystem	17	9	8	0,2%	0,1%	0,4%
X64 Vorsätzliche Selbstvergiftung durch und Exposition gegenüber sonstige(n) und nicht näher bezeichnete(n) Arzneimittel(n), Drogen und biologisch aktive(n) Substanzen	621	324	297	6,9%	4,7%	13,5%
X65 Vorsätzliche Selbstvergiftung durch und Exposition gegenüber Alkohol	22	14	8	0,2%	0,2%	0,4%
X66 Vorsätzliche Selbstvergiftung durch und Exposition gegenüber organische(n) Lösungsmittel(n) oder halogenierte(n) Kohlenwasserstoffe(n) und deren Dämpfe(n)	3	1	2	0,0%	0,0%	0,1%

X67 Vorsätzliche Selbstvergiftung durch und Exposition gegenüber sonstige(n) Gase(n) und Dämpfe(n)	387	326	61	4,3%	4,8%	2,8%
X68 Vorsätzliche Selbstvergiftung durch und Exposition gegenüber Schädlingsbekämpfungsmittel(n) [Pestizide(n)]	21	14	7	0,2%	0,2%	0,3%
X69 Vorsätzliche Selbstvergiftung durch und Exposition gegenüber sonstige(n) oder nicht näher bezeichnete(n) Chemikalien und schädliche(n) Substanzen	149	91	58	1,6%	1,3%	2,6%
X70 Vorsätzliche Selbstbeschädigung durch Erhängen, Strangulierung oder Ersticken	4.074	3.358	716	45,1%	49,1%	32,6%
X71 Vorsätzliche Selbstbeschädigung durch Ertrinken und Untergehen	200	115	85	2,2%	1,7%	3,9%
X72 Vorsätzliche Selbstbeschädigung durch Handfeuerwaffe	108	106	2	1,2%	1,5%	0,1%
X73 Vorsätzliche Selbstbeschädigung durch Gewehr, Schrotflinte oder schwerere Feuerwaffe [Schusswaffe]	42	42	0	0,5%	0,6%	0,0%
X74 Vorsätzliche Selbstbeschädigung durch sonstige oder nicht näher bezeichnete Feuerwaffe [Schusswaffe]	516	497	19	5,7%	7,3%	0,9%
X75 Vorsätzliche Selbstbeschädigung durch Explosivstoffe	5	4	1	0,1%	0,1%	0,0%
X76 Vorsätzliche Selbstbeschädigung durch Rauch, Feuer und Flammen	66	44	22	0,7%	0,6%	1,0%
X77 Vorsätzliche Selbstbeschädigung durch Wasserdampf, heiße Dämpfe oder heiße Gegenstände	1	1	0	0,0%	0,0%	0,0%
X78 Vorsätzliche Selbstbeschädigung durch scharfen Gegenstand	400	314	86	4,4%	4,6%	3,9%

X79 Vorsätzliche Selbstbeschädigung durch stumpfen Gegenstand	1	1	0	0,0%	0,0%	0,0%
X80 Vorsätzliche Selbstbeschädigung durch Sturz in die Tiefe	917	594	323	10,1%	8,7%	14,7%
X81 Vorsätzliche Selbstbeschädigung durch Sichwerfen oder Sichlegen vor ein sich bewegendes Objekt	483	351	132	5,3%	5,1%	6,0%
X82 Vorsätzliche Selbstbeschädigung durch absichtlich verursachten Kraftfahrzeugunfall	81	62	19	0,9%	0,9%	0,9%
X83 Vorsätzliche Selbstbeschädigung auf sonstige näher bezeichnete Art und Weise	106	72	34	1,2%	1,1%	1,5%
X84 Vorsätzliche Selbstbeschädigung auf nicht näher bezeichnete Art und Weise	491	345	146	5,4%	5,0%	6,6%

Quelle: Statistisches Bundesamt, Todesursachenstatistik, 2021
